# Use of Fibrates and Cancer Risk: A Systematic Review and Meta-Analysis of 17 Long-Term Randomized Placebo-Controlled Trials

**DOI:** 10.1371/journal.pone.0045259

**Published:** 2012-09-19

**Authors:** Stefanos Bonovas, Georgios K. Nikolopoulos, Pantelis G. Bagos

**Affiliations:** 1 Department of Pharmacology, School of Medicine, University of Athens, Athens, Greece; 2 Hellenic Centre for Disease Control and Prevention, Athens, Greece; 3 Department of Computer Science and Biomedical Informatics, University of Central Greece, Lamia, Greece; Copenhagen University Hospital, Denmark

## Abstract

**Background:**

Fibrates comprise a class of well-established antilipidemic agents that significantly reduce cardiovascular events. Given the concerns of cancer with fibrate therapy, we undertook a systematic review and meta-analysis to investigate the effects of fibrates on cancer outcomes.

**Methods:**

We systematically searched Medline, Scopus, SCI Expanded, and the Cochrane Library for studies published up to 2012. We included randomized controlled trials (RCTs) that evaluated a fibrate therapy compared with placebo, had a minimum duration of two years, and reported data on the incidence of and/or deaths from cancer during the trial. Reviews of each study were performed and the relative data were abstracted. Pooled relative risk estimates (RR) and 95% confidence intervals (CIs) were calculated using the inverse variance weighted approach. Subgroup, sensitivity and meta-regression analyses were also conducted.

**Results:**

Seventeen RCTs, involving 44,929 participants with an average follow-up of 5.2 years, contributed to the analysis. The degree of variability between trials was consistent with what would be expected to occur by chance alone. The quantitative synthesis of data retrieved from the RCTs was not indicative of a fibrate effect on cancer incidence (780 [fibrate] vs 814 [control]; RR = 1.02, 95% CI 0.92–1.12) or cancer death (385 [fibrate] vs 377 [control]; RR = 1.06, 95% CI: 0.92–1.22). When the analysis was restricted to major RCTs, the results did not substantially change. Similarly, we found no evidence of differential effects by length of follow-up or type of fibrate. Insignificant results were also obtained for the role of fibrates in cancers of the respiratory tract, breast, colon, gastrointestinal tract, prostate, genitourinary tract, or in melanoma.

**Conclusion:**

Our findings demonstrate that fibrates have a neutral effect on cancer outcomes. However, it is important to continue monitoring their long-term safety profiles.

## Introduction

Fibrates comprise an important class of therapeutic agents for the management of dyslipidemia. They are agonists of the peroxisome proliferator-activated receptors alpha (PPAR-*α*), which are mainly expressed in liver, heart and skeletal muscle [1]. Fibrates have been shown to stimulate the expression of genes involved in fatty acid and lipoprotein metabolism, resulting in a shift from hepatic fat synthesis to fat oxidation [2]. This leads to a substantial reduction in serum triglycerides and an increase in high-density lipoprotein cholesterol levels. Though cardiovascular protection using antilipidemic agents has largely been dominated by the 3-hydroxy-3-methylglutaryl coenzyme A reductase inhibitors (statins) since the 1990s, fibrates have been particularly useful in patients with primary hypertriglyceridemia, mixed hyperlipidemia, and in patients with type 2 diabetes and dyslipidemia characterized by high triglyceride and low high-density lipoprotein cholesterol concentrations.

However, in the current era of intensive lipid-lowering therapies to reduce the risk of cardiovascular disease, there is still debate regarding the potential relationship between fibrates use and cancer. Concerns about a possible increase in cancer-related deaths arose initially due to fibrates’ contribution to the total mortality observed during the “in-trial” period of the World Health Organization (WHO) cooperative study [3], although subsequent extended follow-up showed a smaller difference in incidence of and death rates from cancer between treatment and control arms [4], [5]. On the other hand, a review of rodent carcinogenicity tests reported that lipid-lowering drugs, including fibrates, initiate or promote cancer in rats and mice [6]. However, in most of the reviewed studies the doses used were substantially higher than the recommended doses for humans and the employed bioassays were criticised for being inadequate to predict carcinogenicity in humans [7].

Given that the use of fibrates has steadily increased during the past decade [8], more knowledge is needed on the relationship between these medications and cancer. To address this issue, we conducted a systematic review and meta-analysis of randomized placebo-controlled trials published in the peer-reviewed literature.

## Materials and Methods

### Search Strategy

To identify the studies of interest, we systematically searched the following databases: (i) Medline, (ii) Scopus, and (iii) Science Citation Index Expanded, from the date of inception of each database to January 2012. Search terms included: “fibrate” or “fibric acid” or “fenofibrate” or “bezafibrate” or “ciprofibrate” or “clofibrate” or “gemfibrozil”. The search was limited to randomized controlled trials (RCTs), human subjects. The Cochrane Central Register of Controlled Trials was also reviewed. The title and abstract of studies identified in the computerized search were scanned to exclude any that were clearly irrelevant. The full text of the remaining articles was read to determine whether it contained information on the topic of interest. The reference lists of the articles were reviewed to identify citations to other studies of the same topic. No language restrictions were imposed.

### Inclusion and Exclusion Criteria

The studies considered in this meta-analysis were RCTs that evaluated exposure to fibrates and cancer risk. They were considered eligible if they had evaluated a fibrate therapy compared with placebo, they had a minimum duration of two years, and reported data on the incidence of and/or deaths from cancer during the trial. We excluded trials that evaluated multi-interventional therapies where the effect of the fibrate could not be separated out.

We did not assess the methodological quality of the primary studies as quality assessment in meta-analysis is controversial and results can be highly misleading [9], [10]. Instead, we performed subgroup and sensitivity analyses according to study characteristics.

### Data Extraction

Two reviewers (SB, GN) abstracted the data independently. The following information was collected from each study: (i) publication data: first author’s last name, year of publication and geographical location of the study; (ii) study design; (iii) number of participants; (iv) population characteristics; and (v) interventions’ parameters including type of drug, dose and duration. Study-level risk ratios and their 95% confidence intervals (CIs) were estimated by reconstructing contingency tables based on the number of patients randomly assigned and the number of patients who had experienced cancer events (intention-to-treat analysis). When no cancer events occurred in either or both arms, a continuity correction of 0.5 was added to each cell of the respective contingency table. Non-melanoma skin cancers were not included in the analysis because they were neither recorded nor routinely reported in the primary studies. Differences in data extraction were resolved by consensus, referring back to the original article.

### Quantitative Data Synthesis

We used the inverse variance weighted approach to calculate summary effect-estimates. Outcome reporting bias was evaluated using the Begg-Mazumdar adjusted rank correlation test [11] and the Egger regression asymmetry test [12]. To evaluate whether the results of the studies were homogeneous, we used the Cochran’s Q test [13]. We also calculated the quantity I^2^ [14], [15] that describes the percentage variation across studies that is due to heterogeneity rather than chance. We regarded an I^2^ value less than 40% as indicative of “not important heterogeneity” and a value higher than 75% as indicative of “considerable heterogeneity” [16].

Subgroup analyses by fibrate type were also performed to investigate potentially different effects on risk. We, then, conducted a sensitivity analysis by restricting the meta-analysis: (i) to trials with a minimum duration of 5 years, (ii) to trials that enrolled at least 1,000 subjects, and (iii) to major trials that fulfilled both previous criteria. Last, we performed site-specific analyses to evaluate the association between fibrate use and type of cancer diagnosis (cancer subtypes: respiratory, breast, genitourinary, prostate, gastrointestinal, colorectal and melanoma). In those analyses, only RCTs reporting at least one site-specific new cancer diagnosis were included.

We also conducted a meta-regression analysis [17] to investigate the impact of certain study characteristics on the study estimates of relative risk. We first converted all risk ratios by logarithmic transformation to achieve more symmetrical distributions. The natural logarithm of the risk ratio was the dependent variable, and (i) the mean age of participants at enrollment, and (ii) the mean duration of follow-up, were entered as covariates. This analysis was an indirect way to deal with aspects such as the possibility of effect modification by age, and to examine for increasing or decreasing risks with increasing duration of drug use, a feature often associated with causal relationships. We applied a weighted regression model, so that the more precise studies have more influence in the analysis. The weight for each trial was equal to the inverse of the sum of the within-trial variance and the residual between-trial variance, which corresponds to a random effects meta-regression analysis [18]. Estimation of the residual between-trial variance was based on a restricted maximum likelihood method [19].

This work was performed in accordance with the PRISMA statement for the conduct of meta-analyses of intervention studies [20]. For all tests, a probability level lower than 0.05 was considered statistically significant. All statistical tests were two-sided. Stata 9 software was used for the statistical analyses (Stata Corp., College Station, Texas, USA).

## Results

### Search Results

**Figure 1:**
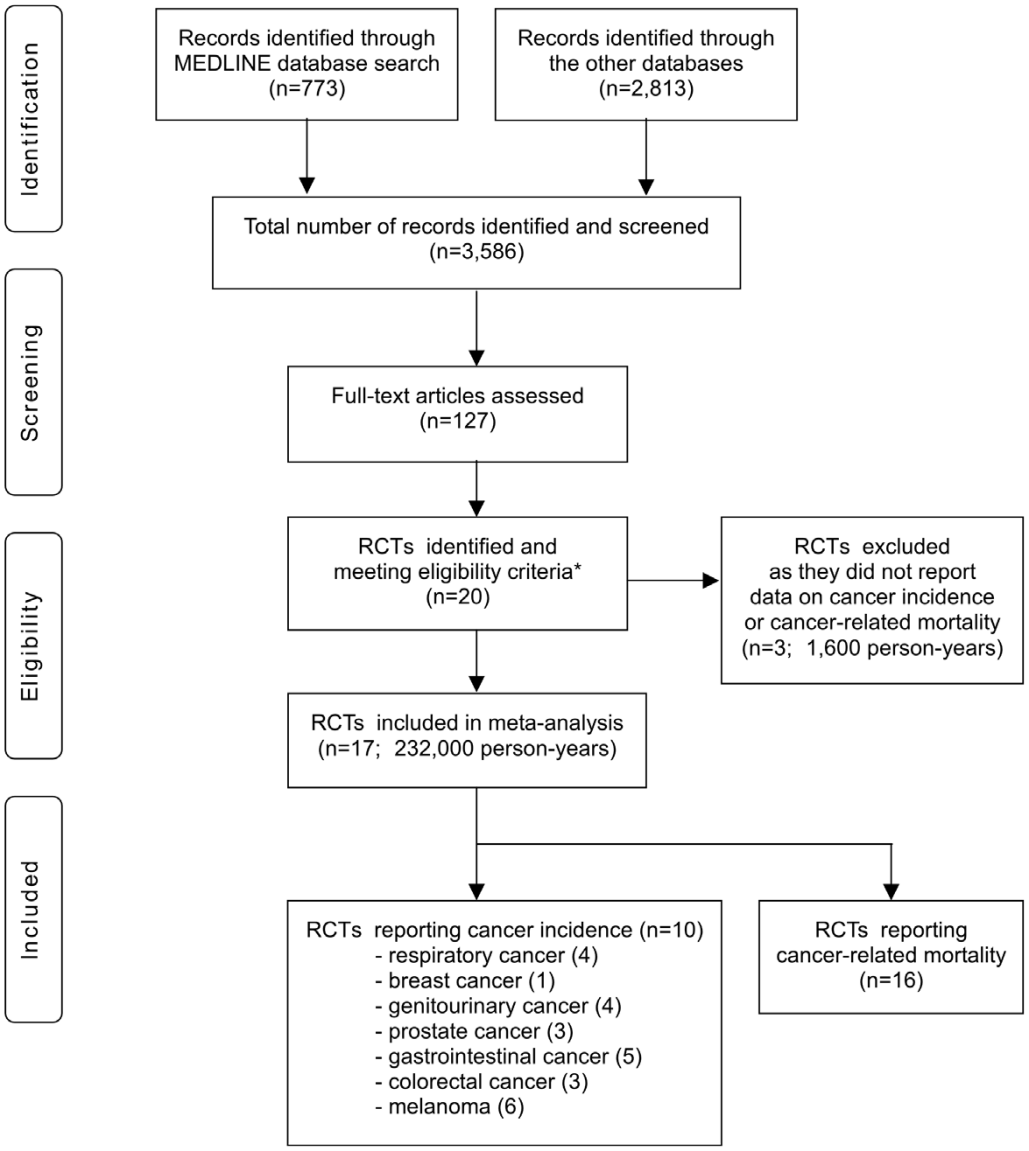
Flow Diagram. Footnote: * To be included in this meta-analysis, studies had to be (i) randomized trials of fibrates, (ii) placebo-controlled, and (iii) have a mean (or median) duration of patient follow-up of at least 2 years.

Our initial search yielded 3,586 literature citations (Figure 1). However, most abstracts were duplicates or did not specifically address the topic of our analysis and were excluded from full-text review. We retrieved 127 potentially relevant manuscripts. The full text was read and the reference lists were checked. We identified 20 studies of fibrates [3], [21]–[39], which conformed to our inclusion criteria. Seventeen of these [3], [21]–[36] evaluated adverse effects and reported data on the incidence of and/or deaths from cancer during the trial, and we were able to conduct post-hoc analyses to calculate risk ratios. For several studies, additional usable data were extracted from other publications [40]–[44]. All 17 studies were randomized placebo-controlled trials, and all but one [23] were double-blind.

A total of 44,929 individuals participated in these trials; 21,627 in treatment groups and 23,302 in placebo groups. The participants had a mean age of 55 years at enrollment, and the average follow-up was 5.2 years. A total of 232,000 person-years were reached. Sixteen RCTs reported data on cancer deaths during the trial [3], [21]–[23], [25]–[36]. The overall cancer mortality was 1.82% (762 cancer deaths during the follow-up) corresponding to a rate of 0.36% per year. On the other hand, 10 RCTs reported data on new cancer diagnoses [3], [22], [24]–[26], [28]–[30], [32], [36]. The overall cancer incidence was 4.48% (1,594 cancer diagnoses) corresponding to a rate of 0.86% per year.

Bezafibrate had been evaluated in four trials [22], [24], [31], [35], clofibrate in six [3], [23], [25], [27], [33], [34], fenofibrate in three [21], [26], [28], and gemfibrozil in four trials [29], [30], [32], [36]. Tables 1 & 2 list the RCTs included in the meta-analysis together with the respective trial drug, the number and summary characteristics of patients, the duration of follow-up and the estimated risk ratios and their 95% CIs.

**Table 1:** Characteristics of studies included in the meta-analysis.

Study	Location	Agent	Design	N	(active/placebo)	Mean age(yrs)	Follow-up (yrs)	Study population
ACCORD [21], 2010	USA, Canada	Fenofibrate	r, db, pc	5518	(2765/2753)	62.2	5.0	Type 2 DM
BECAIT [22], 1996	Sweden	Bezafibrate	r, db, pc	92	(47/45)	42.0	5.0	CAD
Begg Study [23], 1971	Scotland	Clofibrate	r, pc	155	(76/79)	56.0	3.5	Peripheral arteriopathy
BIP [24], 2000	Israel	Bezafibrate	r, db, pc	3090	(1548/1542)	60.1	6.2	CAD
CDP [25], 1975	USA, Puerto Rico	Clofibrate	r, db, pc	3892	(1103/2789)	52.0	6.2	CAD
DAIS [26], 2001	Canada, Finland,France, Sweden	Fenofibrate	r, db, pc	418	(207/211)	56.8	3.3	Type 2 DM
DIS [27], 1991	Germany	Clofibricacid	r, db, pc	761	(379/382)	46.0	5.0	NIDDM
FIELD [28], 2005	Australia,New Zealand, Finland	Fenofibrate	r, db, pc	9795	(4895/4900)	62.2	5.0	Type 2 DM
HHS [29], 1987	Finland	Gemfibrozil	r, db, pc	4081	(2046/2035)	47.3	5.0	Dyslipidemia
HHS ancillary [30],1993	Finland	Gemfibrozil	r, db, pc	628	(311/317)	48.6	5.0	Suspected CAD
LEADER [31], 2002	United Kingdom	Bezafibrate	r, db, pc	1568	(783/785)	68.2	4.6	LEAD
LOCAT [32], 1997	Finland	Gemfibrozil	r, db, pc	395	(197/198)	59.2	2.5	CAD
NEWCASTLE [33], 1971	England	Clofibrate	r, db, pc	497	(244/253)	52.5	3.6	CAD
SCOTTISH [34], 1971	Scotland	Clofibrate	r, db, pc	717	(350/367)	52.1	3.4	CAD
SENDCAP [35], 1998	United Kingdom	Bezafibrate	r, db, pc	164	(81/83)	50.9	3.0	Type 2 DM
VA HIT [36], 1999	USA	Gemfibrozil	r, db, pc	2531	(1264/1267)	64.0	5.1	CAD
WHO [3], 1978	Scotland, Hungary,Czech Rep.	Clofibrate	r, db, pc	10627	(5331/5296)	45.9	5.3	High-cholesterol population

Abbreviations: ACCORD, Action to Control Cardiovascular Risk in Diabetes; BECAIT, Bezafibrate Coronary Atherosclerosis Intervention Trial; BIP, Bezafibrate Infarction Prevention; CDP, Coronary Drug Project; DAIS, Diabetes Atherosclerosis Intervention Study; DIS, Diabetes Intervention Study; FIELD, Fenofibrate Intervention and Event Lowering in Diabetes; HHS, Helsinki Heart Study; LEADER, Lower Extremity Arterial Disease Event Reduction; LOCAT, Lopid Coronary Angiography Trial; SENDCAP, St. Mary’s, Ealing, Northwick Park Diabetes Cardiovascular Disease Prevention; VA HIT, Veterans Affairs High-Density Lipoprotein Cholesterol Intervention Trial; WHO, World Health Organization; r, randomized; db, double-blind; pc, placebo-controlled; DM, Diabetes Mellitus; CAD, Coronary Artery Disease; NIDDM, Non-Insulin-Dependent Diabetes Mellitus; LEAD, Lower Extremity Arterial Disease.

**Table 2:** Incidence of and/or deaths from cancer in the randomized placebo-controlled trials of fibrates.

Study	Incident cancer, n (%*)						Cancer deaths, n (%*)					
	Fibrate		Placebo		RR	95% CI	Fibrate		Placebo		RR	95% CI
ACCORD [21], 2010	–	–	–	–	–	–	57	(2.06)	58	(2.11)	0.98	(0.68–1.40)
BECAIT [22], 1996	1	(2.13)	1	(2.22)	0.96	(0.06–14.85)	0	(0.00)	0	(0.00)	0.96	(0.02–47.30)
Begg Study [23], 1971	–	–	–	–	–	–	0	(0.00)	0	(0.00)	1.04	(0.02–51.71)
BIP [24], 2000	85	(5.49)	91	(5.90)	0.93	(0.70–1.24)	–	–	–	–	–	–
CDP [25], 1975	32	(2.90)	79	(2.83)	1.02	(0.68–1.54)	10	(0.91)	24	(0.86)	1.05	(0.51–2.20)
DAIS [26], 2001	5	(2.42)	7	(3.32)	0.73	(0.23–2.26)	0	(0.00)	3	(1.42)	0.15	(0.01–2.80)**
DIS [27], 1991	–	–	–	–	–	–	2	(0.53)	3	(0.79)	0.67	(0.11–4.00)
FIELD [28], 2005	393	(8.03)	373	(7.61)	1.05	(0.92–1.21)	168	(3.43)	148	(3.02)	1.14	(0.91–1.41)
HHS [29], 1987	25	(1.22)	29	(1.43)	0.86	(0.50–1.46)	10	(0.49)	11	(0.54)	0.90	(0.38–2.12)
HHS ancillary [30], 1993	5	(1.61)	4	(1.26)	1.27	(0.35–4.70)	0	(0.00)	2	(0.63)	0.20	(0.01–4.23)
LEADER [31], 2002	–	–	–	–	–	–	47	(6.00)	47	(5.99)	1.00	(0.68–1.48)
LOCAT [32], 1997	3	(1.52)	7	(3.54)	0.43	(0.11–1.64)	0	(0.00)	0	(0.00)	1.01	(0.02–50.41)
NEWCASTLE [33], 1971	–	–	–	–	–	–	3	(1.23)	1	(0.40)	3.11	(0.33–29.70)
SCOTTISH [34], 1971	–	–	–	–	–	–	3	(0.86)	5	(1.36)	0.63	(0.15–2.61)
SENDCAP [35], 1998	–	–	–	–	–	–	0	(0.00)	0	(0.00)	1.02	(0.02–51.02)
VA HIT [36], 1999	125	(9.89)	138	(10.89)	0.91	(0.72–1.14)	45	(3.56)	51	(4.03)	0.88	(0.60–1.31)
WHO [3], 1978	106	(1.99)	85	(1.60)	1.24	(0.93–1.64)	40	(0.75)	24	(0.45)	1.66	(1.00–2.74)

* Crude rates;

** Reported in Saha et al. [43].

Abbreviations: ACCORD, Action to Control Cardiovascular Risk in Diabetes; BECAIT, Bezafibrate Coronary Atherosclerosis Intervention Trial; BIP, Bezafibrate Infarction Prevention; CDP, Coronary Drug Project; DAIS, Diabetes Atherosclerosis Intervention Study; DIS, Diabetes Intervention Study; FIELD, Fenofibrate Intervention and Event Lowering in Diabetes; HHS, Helsinki Heart Study; LEADER, Lower Extremity Arterial Disease Event Reduction; LOCAT, Lopid Coronary Angiography Trial; SENDCAP, St. Mary’s, Ealing, Northwick Park Diabetes Cardiovascular Disease Prevention; VA HIT, Veterans Affairs High-Density Lipoprotein Cholesterol Intervention Trial; WHO, World Health Organization; RR, relative risk (risk ratio); CI, confidence interval.

### Meta-analysis of Fibrate Use and Cancer Mortality

**Figure 2:**
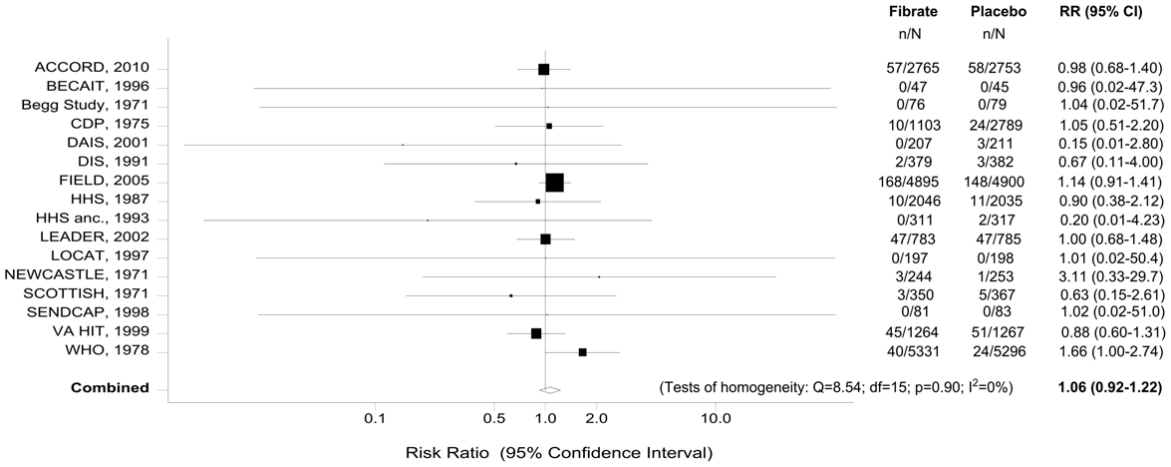
Forest plot of the meta-analysis of fibrate use and cancer deaths. Footnote: The risk ratios and their 95% confidence intervals are displayed on a logarithmic scale. The size of the data markers represents the relative weight of the trial according to size and occurrence of the outcome being measured.

Sixteen RCTs [3], [21]–[23], [25]–[36] reported data on cancer related-deaths. The meta-analysis of these trials showed no evidence of an association between fibrate therapy and cancer mortality (RR = 1.06, 95% CI: 0.92–1.22) (Table 3). Figure 2 graphs the risk ratios and the corresponding 95% CIs from the individual studies, and the pooled results. The Cochran’s Q test had a p-value of 0.90 and the corresponding I^2^ was 0%, which both indicate very small between-studies variability (Table 3). The p-values for the Begg’s and the Egger’s tests were p = 0.89 and p = 0.18, respectively, both suggesting that the assumption of no reporting bias is reasonable.

**Table 3:** Fibrate use and cancer risk: Meta-analysis and subgroup analysis.

		Pooled effect estimate		Tests of homogeneity			Tests for reporting bias	
	N	RR	(95% CI)	Q (d.f.)	p-value	I^2^	Begg’s p	Egger’s p
**Cancer Deaths:**								
All RCTs	16	1.06	(0.92–1.22)	8.54 (15)	0.90	0%	0.89	0.18
Bezafibrate	3	1.00	(0.68–1.48)	0.00 (2)	0.99	0%	0.99	0.79
Clofibrate	6	1.31	(0.90–1.92)	2.88 (5)	0.72	0%	0.99	0.32
Fenofibrate	3	1.08	(0.90–1.31)	2.26 (2)	0.32	12%	0.30	0.077
Gemfibrozil	4	0.87	(0.61–1.24)	0.90 (3)	0.83	0%	0.31	0.43
Duration ≥5 years	9	1.08	(0.93–1.26)	5.85 (8)	0.66	0%	0.75	0.34
Participants ≥1,000	7	1.08	(0.94–1.24)	4.55 (6)	0.60	0%	0.99	0.84
Both previous criteria	6	1.09	(0.94–1.27)	4.40 (5)	0.49	0%	0.99	0.89
**Cancer Incidence:**								
All RCTs	10	1.02	(0.92–1.12)	5.89 (9)	0.75	0%	0.72	0.28
Bezafibrate	2	0.93	(0.70–1.24)	0.00 (1)	0.98	0%	0.99	–
Clofibrate	2	1.16	(0.92–1.47)	0.57 (1)	0.45	0%	0.99	–
Fenofibrate	2	1.05	(0.92–1.20)	0.41 (1)	0.52	0%	0.99	–
Gemfibrozil	4	0.89	(0.73–1.10)	1.47 (3)	0.69	0%	0.73	0.64
Duration ≥5 years	8	1.02	(0.93–1.13)	3.95 (7)	0.79	0%	0.99	0.81
Participants ≥1,000	6	1.02	(0.93–1.13)	3.84 (5)	0.57	0%	0.99	0.60
Both previous criteria	6	1.02	(0.93–1.13)	3.84 (5)	0.57	0%	0.99	0.60
Respiratory Cancer	4	0.99	(0.71–1.37)	1.36 (3)	0.72	0%	0.99	0.92
Breast Cancer	1	0.98	(0.63–1.53)	–	–	–	–	–
Genitourinary Cancer	4	1.11	(0.89–1.38)	2.05 (3)	0.56	0%	0.73	0.89
Prostate Cancer	3	1.25	(0.96–1.63)	1.24 (2)	0.54	0%	0.99	0.99
Gastrointestinal Cancer	5	0.91	(0.73–1.14)	5.29 (4)	0.26	24%	0.46	0.033
Colorectal Cancer	3	0.98	(0.71–1.34)	3.96 (2)	0.14	49%	0.99	0.43
Melanoma	6	0.54	(0.22–1.31)	5.50 (5)	0.36	9%	0.99	0.66

RR, risk ratio; CI, confidence interval; d.f., degrees of freedom.

**Figure 3:**
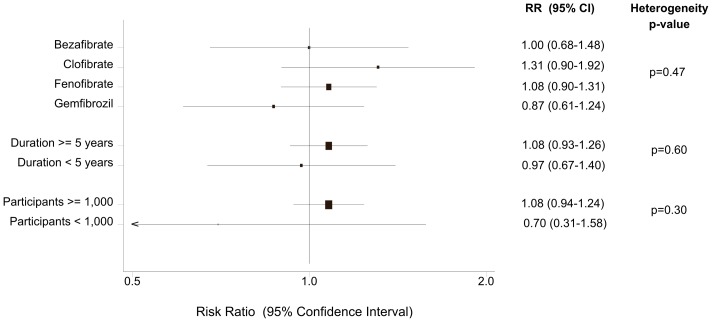
Fibrate use and cancer deaths: Subgroup analyses.

After stratifying the data into subgroups, according to the type of fibrate, we did not find any statistically significant association between bezafibrate, clofibrate, fenofibrate or gemfibrozil use and cancer mortality (Table 3, Figure 3).

When the analysis was restricted to studies with a minimum duration of 5 years (9 trials), we also found no evidence of an association (RR = 1.08, 95% CI: 0.93–1.26). Similarly, when only RCTs that enrolled at least 1,000 subjects contributed to the analysis (7 trials), the pooled effect estimate remained insignificant (RR = 1.08, 95% CI: 0.94–1.24) (Table 3, Figure 3). Finally, we performed a meta-analysis of six major trials [3], [21], [25], [28], [29], [36] that fulfilled both previous criteria. Once again, there was no statistically significant association between fibrate use and cancer mortality (RR = 1.09, 95% CI: 0.94–1.27). In this restricted analysis, there was no evidence of reporting bias (Begg’s p = 0.99, Egger’s p = 0.89) or heterogeneity (Cochran’s Q test: p = 0.49, I^2^ = 0%) (Table 3).

Meta-regression analysis, using the mean age of participants and the mean duration of follow-up as covariates, did not reveal any significant association (Table 4).

**Table 4:** Meta-regression’s results.

	Univariable analysis		
	Ratio of RR	(95% CI)	p-value
**Cancer Deaths**:			
- Mean age of participants (per 10-year increase)	0.89	(0.71–1.12)	0.33
- Mean duration of follow-up (per 1-year increase)	1.19	(0.81–1.74)	0.37
**Cancer Incidence**:			
- Mean age of participants (per 10-year increase)	0.92	(0.79–1.08)	0.33
- Mean duration of follow-up (per 1-year increase)	1.03	(0.85–1.25)	0.73

RR, risk ratio; CI, confidence interval.

Results are exponentiated regression coefficients and their 95% CIs, which show the proportional change in risk ratio for every one scale increase.

### Meta-analysis of Fibrate Use and Cancer Incidence

**Figure 4:**
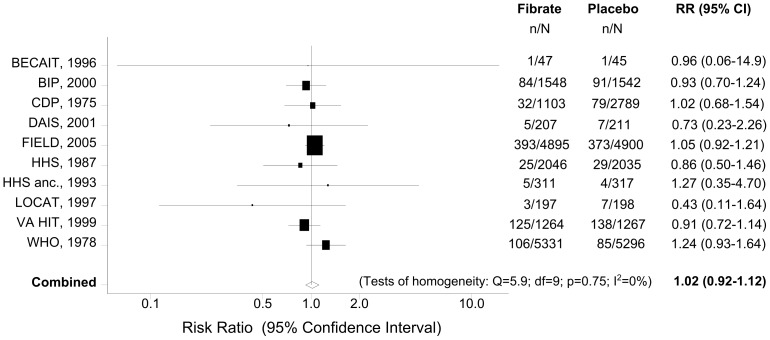
Forest plot of the meta-analysis of fibrate use and cancer incidence. Footnote: The risk ratios and their 95% confidence intervals are displayed on a logarithmic scale. The size of the data markers represents the relative weight of the trial according to size and occurrence of the outcome being measured.

Ten RCTs [3], [22], [24]–[26], [28]–[30], [32], [36] reported data on new cancer diagnoses. The quantitative synthesis of these studies provided no evidence of an association between fibrate use and cancer incidence. The calculated effect estimate indicated a neutral effect of fibrates (RR = 1.02, 95% CI: 0.92–1.12) (Table 3). Figure 4 presents a forest plot of the risk ratios and their 95% CIs from the 10 primary studies, and the pooled estimates. Reporting bias (Begg’s p = 0.72; Egger’s p = 0.28) or significant heterogeneity (Cochran’s p = 0.75; I^2^ = 0%) were not detected in this analysis (Table 3).

**Figure 5:**
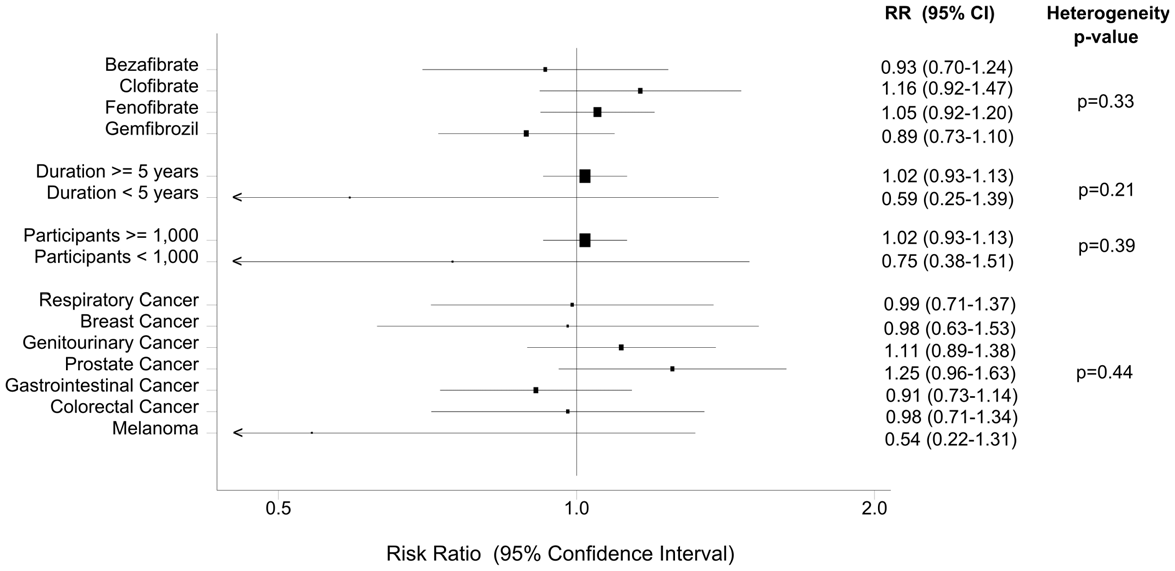
Fibrate use and cancer incidence: Subgroup analyses.

The subgroup analysis, according to the type of fibrate, did not demonstrate any significant association between bezafibrate, clofibrate, fenofibrate or gemfibrozil use and cancer incidence (Table 3, Figure 5).

When we restricted the analysis to studies with a minimum duration of 5 years (8 trials), we also found no evidence of association (RR = 1.02, 95% CI: 0.93–1.13). Similarly, when only RCTs with a minimum duration of 5 years and more than 1,000 participants (6 trials) [3], [24], [25], [28], [29], [36] contributed to the analysis, the summary effect estimate suggested a neutral effect of fibrates on cancer incidence (RR = 1.02, 95% CI: 0.93–1.13; Begg’s p = 0.99; Egger’s p = 0.60; Cochran’s p = 0.57; I^2^ = 0%) (Table 3, Figure 5).

No statistically significant differences were observed between patients receiving fibrate vs placebo for any of the prespecified cancer subtypes (Table 3, Figure 5). Heterogeneity or reporting bias were not observed in any of these site-specific analyses (p>0.14 for all) with the exception of gastrointestinal cancer (Egger’s p = 0.033; Table 3).

Last, in the meta-regression analysis, the results did not show any impact of either the mean age of participants or the duration of follow-up on the study estimates of relative risk (Table 4).

In a re-analysis, excluding all trials reporting zero cancer events in either or both arms, no statistically or clinically meaningful differences occurred vs the initial analyses (both for cancer mortality and cancer incidence).

## Discussion

Fibrates comprise a class of lipid-lowering agents that significantly reduce cardiovascular events through a substantial reduction in serum levels of triglycerides and modest effects on levels of low-density and high-density lipoprotein cholesterol [45], [46]. The concerns that lipid-lowering drugs (including fibrates) might increase the risk of cancer have been present for three decades [6], [47]–[51]. However, while for the 3-hydroxy-3-methylglutaryl coenzyme A reductase inhibitors (statins) several meta-analyses of existing data have convincingly demonstrated that they do not cause any substantial change in overall [52], [53] or site-specific cancer risk [54]–[59], this is the first meta-analysis that focuses specifically on the relationship between fibrates and cancer.

Our study encompassed 17 randomized placebo-controlled trials involving 45,000 individuals with a broad range of baseline characteristics, and accrued a total experience of 232,000 person-years. The analysis did not provide any evidence that the use of fibrates significantly affects the incidence of cancer or cancer-related death. When the analysis was restricted to major RCTs, the results remained practically unchanged. Similarly, when bezafibrate, clofibrate, fenofibrate and gemfibrozil were evaluated alone, we found no impact on cancer outcomes. No significant results were also noted for cancers of the respiratory tract, breast, colon, gastrointestinal tract, prostate, genitourinary tract, or skin (melanoma) when fibrates were used.

The neutral results of the present study are in line with several well-designed observational studies that have analysed overall cancer [60], [61] and site-specific cancers [62]–[64] in fibrate users, and exclude the strong protective effect of fibrates found in the PRIME study [65]. This large observational cohort, suggesting that cancer mortality was significantly lower in fibrate users as compared with untreated dyslipidemic subjects (hazard ratio: 0.52, 95% CI: 0.28–0.97), may have been affected by uncontrolled (unmeasured) or residual confounding or other biases, problems known to plague even well-designed observational studies because they lack the experimental random allocation of the intervention necessary to test exposure-outcome hypotheses [66].

Our study has several merits. We have conducted an extensive literature search to retrieve all relevant eligible trials. Moreover, the absence of significant between-study heterogeneity, the small likelihood of important reporting bias, as well as the stability of results in subgroup and sensitivity analyses, reinforce our confidence in the validity of the conclusion that fibrate use has a neutral effect on cancer outcomes. The strengths of this quantitative synthesis should be, however, weighed against some limitations. First, the trials included in this meta-analysis were not designed to specifically analyze the relationship between fibrates and cancer risk. They have assessed cancer outcomes as secondary (safety) endpoints. Thus, problems in cancer detection and reporting may exist. However, the definition used and the surveillance intensity were consistent within each study for the fibrate and placebo groups, so the relative impact should still be accurate. Second, our search was restricted to published studies and we did not seek for unpublished/original data. However, we did not impose any exclusion criteria with regard to language, place of publication or study quality. Last, a main issue remaining beyond our control is cancer latency. As the exposure and follow-up times only lasted for nearly five years, estimates of cancer risk resulting from longer exposure to fibrates are not possible. Thus, our results should be interpreted with caution.

In conclusion, the synthesis of existing data from randomized placebo-controlled trials supports a neutral effect of fibrates on cancer risk in the short term. We found no site-specific type of cancer whose risk was affected by fibrates, or subtype of fibrates that influenced the risk of cancer. However, given the steadily increasing use of fibrates during the past decade [8], and the indications for long-term and perhaps lifelong use, it is important to continue monitoring their long-term safety profiles.

## Acknowledgments

### Data Access and Responsibility

Stefanos Bonovas had full access to all the data in the study, takes responsibility for the integrity of the data and the accuracy of the data analysis and acts as guarantor of the paper.

## References

[1] Staels B, Fruchart JC (2005) Therapeutic roles of peroxisome proliferator-activated receptor agonists. Diabetes 54: 2460–2470. 16046315 10.2337/diabetes.54.8.2460

[2] Després JP, Lemieux I, Robins SJ (2004) Role of fibric acid derivatives in the management of risk factors for coronary heart disease. Drugs 64: 2177–2198. 15456334 10.2165/00003495-200464190-00003

[3] WHO Cooperative Trial Committee of Principal Investigators (1978) A cooperative trial in the primary prevention of ischaemic heart disease using clofibrate. Br Heart J 40: 1069–1118. 361054 10.1136/hrt.40.10.1069 PMC483536

[4] WHO Cooperative Trial Committee of Principal Investigators (1985) WHO cooperative trial on primary prevention of ischaemic heart disease using clofibrate to lower serum cholesterol: mortality follow-up. Lancet 2: 379–385. 6105515

[5] Heady JA, Morris JN, Oliver MF (1992) WHO clofibrate/cholesterol trial: clarifications. Lancet 340: 1405–1406. 1360101 10.1016/0140-6736(92)92588-7

[6] Newman TB, Hulley SB (1996) Carcinogenicity of lipid-lowering drugs. JAMA 275: 55–60. 8531288

[7] Dalen JE, Dalton WS (1996) Does lowering cholesterol cause cancer? JAMA 275: 67–69. 8531290

[8] Jackevicius CA, Tu JV, Ross JS, Ko DT, Carreon D, et al (2011) Use of fibrates in the United States and Canada. JAMA 305: 1217–1224. 21427374 10.1001/jama.2011.353 PMC3332101

[9] Greenland S (1994) Invited commentary: a critical look at some popular meta-analytic methods. Am J Epidemiol 140: 290–296. 8030632 10.1093/oxfordjournals.aje.a117248

[10] Jüni P, Witschi A, Bloch R, Egger M (1999) The hazards of scoring the quality of clinical trials for meta-analysis. JAMA 282: 1054–1060. 10493204 10.1001/jama.282.11.1054

[11] Begg CB, Mazumdar M (1994) Operating characteristics of a rank correlation test for publication bias. Biometrics 50: 1088–1101. 7786990

[12] Egger M, Davey Smith G, Schneider M, Minder C (1997) Bias in meta-analysis detected by a simple, graphical test. BMJ 315: 629–634. 9310563 10.1136/bmj.315.7109.629 PMC2127453

[13] Cochran WG (1954) The combination of estimates from different experiments. Biometrics 8: 101–129.

[14] Higgins JP, Thompson SG (2002) Quantifying heterogeneity in a meta-analysis. Stat Med 21: 1539–1558. 12111919 10.1002/sim.1186

[15] Higgins JP, Thompson SG, Deeks JJ, Altman DG (2003) Measuring inconsistency in meta-analyses. BMJ 327: 557–560. 12958120 10.1136/bmj.327.7414.557 PMC192859

[16] Higgins JPT, Green S (2008) Cochrane handbook for systematic reviews of interventions. The Cochrane Collaboration.

[17] Sharp S (1998) Meta-analysis regression. Stata Tech Bull 42: 16–24.

[18] Berkey CS, Hoaglin DC, Mosteller F, Colditz GA (1995) A random-effects regression model for meta-analysis. Stat Med 14: 395–411. 7746979 10.1002/sim.4780140406

[19] Thompson SG, Sharp SJ (1999) Explaining heterogeneity in meta-analysis: a comparison of methods. Stat Med 18: 2693–2708. 10521860 10.1002/(sici)1097-0258(19991030)18:20<2693::aid-sim235>3.0.co;2-v

[20] Moher D, Liberati A, Tetzlaff J, Altman DG (2009) Preferred reporting items for systematic reviews and meta-analyses: the PRISMA statement. Ann Intern Med 151: 264–269, W64. 10.7326/0003-4819-151-4-200908180-00135 19622511

[21] Ginsberg HN, Elam MB, Lovato LC, Crouse JR 3rd, Leiter LA (2010) Effects of combination lipid therapy in type 2 diabetes mellitus. N Engl J Med 362: 1563–1574. 20228404 10.1056/NEJMoa1001282 PMC2879499

[22] Ericsson CG, Hamsten A, Nilsson J, Grip L, Svane B, et al (1996) Angiographic assessment of effects of bezafibrate on progression of coronary artery disease in young male postinfarction patients. Lancet 347: 849–853. 8622389 10.1016/s0140-6736(96)91343-4

[23] Begg TB, Rifkind BM (1971) Evaluation of clofibrate therapy in peripheral arteriopathy. Minerva Med 62: 3469–3475. 5097219

[24] The BIP Study Group (2000) Secondary prevention by raising HDL cholesterol and reducing triglycerides in patients with coronary artery disease: the Bezafibrate Infarction Prevention (BIP) study. Circulation 102: 21–27. 10880410 10.1161/01.cir.102.1.21

[25] The Coronary Drug Project Research Group (1975) Clofibrate and niacin in coronary heart disease. JAMA 231: 360–381. 1088963

[26] Diabetes Atherosclerosis Intervention Study Investigators (2001) Effect of fenofibrate on progression of coronary-artery disease in type 2 diabetes: the Diabetes Atherosclerosis Intervention Study, a randomised study. Lancet 357: 905–910. 11289345

[27] Hanefeld M, Fischer S, Schmechel H, Rothe G, Schulze J, et al (1991) Diabetes Intervention Study. Multi-intervention trial in newly diagnosed NIDDM. Diabetes Care 14: 308–317. 2060433 10.2337/diacare.14.4.308

[28] Keech A, Simes RJ, Barter P, Best J, Scott R, et al (2005) Effects of long-term fenofibrate therapy on cardiovascular events in 9795 people with type 2 diabetes mellitus (the FIELD study): randomised controlled trial. Lancet 366: 1849–1861. 16310551 10.1016/S0140-6736(05)67667-2

[29] Huttunen JK, Heinonen OP, Manninen V, Koskinen P, Hakulinen T, et al (1994) The Helsinki Heart Study: an 8.5-year safety and mortality follow-up. J Intern Med 235: 31–39. 8283157 10.1111/j.1365-2796.1994.tb01029.x

[30] Frick MH, Heinonen OP, Huttunen JK, Koskinen P, Mänttäri M, et al (1993) Efficacy of gemfibrozil in dyslipidaemic subjects with suspected heart disease. An ancillary study in the Helsinki Heart Study frame population. Ann Med 25: 41–45. 8435186 10.3109/07853899309147855

[31] Meade T, Zuhrie R, Cook C, Cooper J (2002) Bezafibrate in men with lower extremity arterial disease: randomised controlled trial. BMJ 325: 1139. 12433762 10.1136/bmj.325.7373.1139 PMC133451

[32] Frick MH, Syvänne M, Nieminen MS, Kauma H, Majahalme S, et al (1997) Prevention of the angiographic progression of coronary and vein-graft atherosclerosis by gemfibrozil after coronary bypass surgery in men with low levels of HDL cholesterol. Lopid Coronary Angiography Trial (LOCAT) Study Group. Circulation 96: 2137–2143. 9337181 10.1161/01.cir.96.7.2137

[33] Group of physicians of the Newcastle upon Tyne region (1971) Trial of clofibrate in the treatment of ischaemic heart disease. Five-year study. Br Med J 4: 767–775. 4943605 PMC1799703

[34] Research Committee of the Scottish Society of Physicians (1971) Ischaemic heart disease: a secondary prevention trial using clofibrate. Br Med J 4: 775–784. 4943606 PMC1799730

[35] Elkeles RS, Diamond JR, Poulter C, Dhanjil S, Nicolaides AN, et al (1998) Cardiovascular outcomes in type 2 diabetes. A double-blind placebo-controlled study of bezafibrate: the St. Mary’s, Ealing, Northwick Park Diabetes Cardiovascular Disease Prevention (SENDCAP) Study. Diabetes Care 21: 641–648. 9571357 10.2337/diacare.21.4.641

[36] Rubins HB, Robins SJ, Collins D, Fye CL, Anderson JW, et al (1999) Gemfibrozil for the secondary prevention of coronary heart disease in men with low levels of high-density lipoprotein cholesterol. N Engl J Med 341: 410–418. 10438259 10.1056/NEJM199908053410604

[37] Acheson J, Hutchinson EC (1972) Controlled trial of clofibrate in cerebral vascular disease. Atherosclerosis 15: 177–183. 4579955 10.1016/0021-9150(72)90067-6

[38] Cullen JF, Town SM, Campbell CJ (1972) Double-blind trial of Atromid-S in exudative diabetic retinopathy. Trans Ophthalmol Soc UK 94: 554–562. 4619857

[39] The Veterans Administration Cooperative Study Group (1973) The treatment of cerebrovascular disease with clofibrate. Stroke 4: 684–693. 4723698 10.1161/01.str.4.4.684

[40] The Coronary Drug Project Research Group (1978) Estrogens and cancer. JAMA 239: 2758–2759.349183

[41] Dewar HA (1972) Trial of clofibrate. Br Med J 2: 228.

[42] Freeman SR, Drake AL, Heilig LF, Graber M, McNealy K, et al (2006) Statins, fibrates, and melanoma risk: a systematic review and meta-analysis. J Natl Cancer Inst 98: 1538–1546. 17077356 10.1093/jnci/djj412

[43] Saha SA, Arora RR (2010) Fibrates in the prevention of cardiovascular disease in patients with type 2 diabetes mellitus: a pooled meta-analysis of randomized placebo-controlled clinical trials. Int J Cardiol 141: 157–166. 19232762 10.1016/j.ijcard.2008.11.211

[44] Tenenbaum A, Boyko V, Fisman EZ, Goldenberg I, Adler Y, et al (2008) Does the lipid-lowering peroxisome proliferator-activated receptors ligand bezafibrate prevent colon cancer in patients with coronary artery disease? Cardiovasc Diabetol 7: 18. 18565233 10.1186/1475-2840-7-18 PMC2440374

[45] Abourbih S, Filion KB, Joseph L, Schiffrin EL, Rinfret S, et al (2009) Effect of fibrates on lipid profiles and cardiovascular outcomes: a systematic review. Am J Med 122: 962.e1–8. 19698935 10.1016/j.amjmed.2009.03.030

[46] Jun M, Foote C, Lv J, Neal B, Patel A, et al (2010) Effects of fibrates on cardiovascular outcomes: a systematic review and meta-analysis. Lancet 375: 1875–1884. 20462635 10.1016/S0140-6736(10)60656-3

[47] Muldoon MF, Manuck SB, Matthews KA (1990) Lowering cholesterol concentrations and mortality: a quantitative review of primary prevention trials. BMJ 301: 309–314. 2144195 10.1136/bmj.301.6747.309 PMC1663605

[48] Oliver MF (1991) Might treatment of hypercholesterolaemia increase non-cardiac mortality? Lancet 337: 1529–1531. 1675380 10.1016/0140-6736(91)93208-q

[49] Hulley SB, Walsh JM, Newman TB (1992) Health policy on blood cholesterol. Time to change directions. Circulation 86: 1026–1029. 1516172 10.1161/01.cir.86.3.1026

[50] Davey Smith G, Pekkanen J (1992) Should there be a moratorium on the use of cholesterol lowering drugs? BMJ 304: 431–434. 1532138 10.1136/bmj.304.6824.431 PMC1881265

[51] Pedersen T (2009) Lipid-lowering drugs and risk for cancer. Curr Atheroscler Rep 11: 350–357. 19664378 10.1007/s11883-009-0053-3

[52] Bonovas S, Filioussi K, Tsavaris N, Sitaras NM (2006) Statins and cancer risk: a literature-based meta-analysis and meta-regression analysis of 35 randomized controlled trials. J Clin Oncol 24: 4808–4817. 17001070 10.1200/JCO.2006.06.3560

[53] Emberson JR, Kearney PM, Blackwell L, Newman C, Reith C, et al (2012) Lack of effect of lowering LDL cholesterol on cancer: meta-analysis of individual data from 175,000 people in 27 randomised trials of statin therapy. PLoS One 7: e29849. 22276132 10.1371/journal.pone.0029849 PMC3261846

[54] Bonovas S, Filioussi K, Tsavaris N, Sitaras NM (2005) Use of statins and breast cancer: a meta-analysis of seven randomized clinical trials and nine observational studies. J Clin Oncol 23: 8606–8612. 16260694 10.1200/JCO.2005.02.7045

[55] Bonovas S, Filioussi K, Flordellis CS, Sitaras NM (2007) Statins and the risk of colorectal cancer: a meta-analysis of 18 studies involving more than 1.5 million subjects. J Clin Oncol 25: 3462–3468. 17687150 10.1200/JCO.2007.10.8936

[56] Bonovas S, Nikolopoulos G, Filioussi K, Peponi E, Bagos P, et al (2010) Can statin therapy reduce the risk of melanoma? A meta-analysis of randomized controlled trials. Eur J Epidemiol 25: 29–35. 19844794 10.1007/s10654-009-9396-x

[57] Bonovas S, Filioussi K, Sitaras NM (2008) Statins are not associated with a reduced risk of pancreatic cancer at the population level, when taken at low doses for managing hypercholesterolemia: evidence from a meta-analysis of 12 studies. Am J Gastroenterol 103: 2646–2651. 18684187 10.1111/j.1572-0241.2008.02051.x

[58] Bonovas S, Filioussi K, Sitaras NM (2008) Statin use and the risk of prostate cancer: a meta-analysis of 6 randomized clinical trials and 13 observational studies. Int J Cancer 123: 899–904. 18491405 10.1002/ijc.23550

[59] Bonovas S, Filioussi K, Tsantes A, Sitaras NM (2007) Use of statins and risk of haematological malignancies: a meta-analysis of six randomized clinical trials and eight observational studies. Br J Clin Pharmacol 64: 255–262. 17578480 10.1111/j.1365-2125.2007.02959.x PMC2000657

[60] Olsen J, Johansen C, Sørensen H, McLaughlin J, Mellemkjaer L, et al (1999) Lipid-lowering medication and risk of cancer. J Clin Epidemiol 52: 167–169. 10201659 10.1016/s0895-4356(98)00147-4

[61] Blais L, Desgagné A, LeLorier J (2000) 3-Hydroxy-3-methylglutaryl coenzyme A reductase inhibitors and the risk of cancer: a nested case-control study. Arch Intern Med 160: 2363–2368. 10927735 10.1001/archinte.160.15.2363

[62] Murtola TJ, Tammela TL, Lahtela J, Auvinen A (2007) Cholesterol-lowering drugs and prostate cancer risk: a population-based case-control study. Cancer Epidemiol Biomarkers Prev 16: 2226–2232. 18006910 10.1158/1055-9965.EPI-07-0599

[63] Fortuny J, de Sanjosé S, Becker N, Maynadié M, Cocco PL, et al (2006) Statin use and risk of lymphoid neoplasms: results from the European Case-Control Study EPILYMPH. Cancer Epidemiol Biomarkers Prev 15: 921–925. 16702371 10.1158/1055-9965.EPI-05-0866

[64] Poynter JN, Gruber SB, Higgins PD, Almog R, Bonner JD, et al (2005) Statins and the risk of colorectal cancer. N Engl J Med 352: 2184–2192. 15917383 10.1056/NEJMoa043792

[65] Gardette V, Bongard V, Dallongeville J, Arveiler D, Bingham A, et al (2009) Ten-year all-cause mortality in presumably healthy subjects on lipid-lowering drugs (from the Prospective Epidemiological Study of Myocardial Infarction [PRIME] prospective cohort). Am J Cardiol 103: 381–386. 19166693 10.1016/j.amjcard.2008.09.092

[66] Davey Smith G, Ebrahim S (2002) Data dredging, bias, or confounding. BMJ 325: 1437–1438. 12493654 10.1136/bmj.325.7378.1437 PMC1124898

